# Acute Methotrexate Toxicity: A Fatal Condition in Two Cases of Psoriasis

**DOI:** 10.1155/2014/946716

**Published:** 2014-09-08

**Authors:** Pankti Jariwala, Vinay Kumar, Khyati Kothari, Sejal Thakkar, Dipak Dayabhai Umrigar

**Affiliations:** ^1^Department of Skin & VD, Government Medical College & New Civil Hospital, Surat, Gujarat 395001, India; ^2^Department of Skin & VD, GMERS Medical College & General Hospital, Gotri 202, Wings Ville 41, Arunoday Society, Alkapuri, Vadodara, Gujarat 390001, India

## Abstract

We describe two fatal cases of low dose methotrexate (MTX) toxicity in patients with psoriasis, emphasizing the factors that exacerbate MTX toxicity. The first patient was a 50-year-old male of psoriasis on intermittent treatment with MTX. After a treatment-free period of six months, he had self-medication of MTX along with analgesic for joint pain for one week which followed ulceration of the lesions, bone marrow suppression, and eventually death. The second patient was a 37-year-old male of psoriasis, who has taken MTX one week earlier without prior investigations. He had painful ulcerated skin lesions and bone marrow suppression. On investigations, he showed high creatinine level and atrophied, nonfunctioning right kidney on ultrasonography. In spite of dialysis, he succumbed to death. MTX is safe and effective if monitored properly, but inadvertent use may lead to even death also. Prior workup and proper counseling regarding the drug interactions as well as self-medication should be enforced.

## 1. Introduction

Methotrexate (MTX), when used in low doses, has anti-inflammatory and immunosuppressive action. Low dose MTX is an effective and safe treatment for psoriasis being used for more than 50 years [1]. Renal excretion is the primary route of elimination and is dependent upon dosage and route of administration [2]. It is also affected by concomitant ingestion of certain drugs which are protein bound like nonsteroidal anti-inflammatory drugs (NSAIDs), sulfonamides, and barbiturates. This demands careful monitoring of renal function tests and blood counts, along with carefully looking for mucosal lesions or ulcerations in skin to identify acute MTX toxicity. Failure to adhere to guidelines may lead to severe toxicity, even death. There are few publications mentioning adverse reactions of MTX, but very few are there mentioning fatality because of such a safe drug. Here, we report two cases that died of MTX toxicity because of just not abiding by the standard protocol.

## 2. Case Reports

### 2.1. Case 1

A 50-year-old male presented with generalized skin lesions with ulcerations along with erosions over lips and oral cavity and difficulty in swallowing for 2 days. He also had fever with chills.

**Figure 1:**
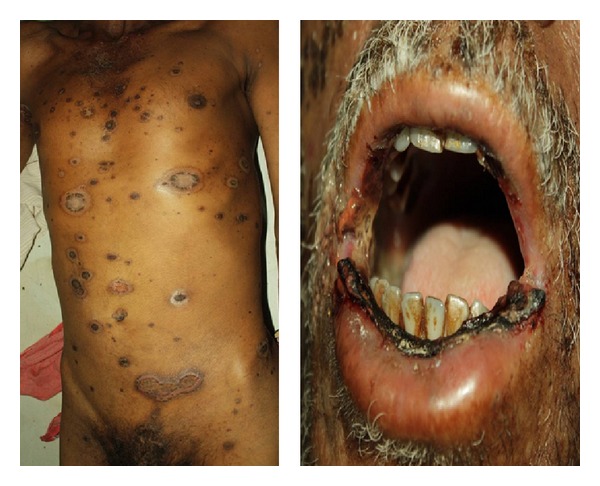
Ulceration over psoriatic lesions with crusting on lips (Case 1).

He was a known case of psoriasis for 5 years, on MTX (7.5 mg) once weekly for two years. He was under remission and stopped MTX six months earlier. He had aggravation of lesions along with knee joint pains for two weeks. He took oral MTX (7.5 mg/day) daily for one week along with some pain killers by himself. After two days he developed ulcerations over existing lesions along with erosions on lips and oral cavity (Figure 1).

The patient was conscious with body temperature 103°F, pulse rate 120/minute, and normal respiration and blood pressure. Cutaneous examination revealed generalized multiple annular ulcerated plaques with mucosal erosions. On admission, investigations showed myelosuppression (Hb 6.7 gms, WBC 1200, and 69,000 platelet count) with normal renal and liver profile.

Patient was diagnosed as a case of acute MTX toxicity and treated with intravenous antibiotics and leucovorin and neukine (GM-CSF) injection subcutaneously. He was investigated periodically which showed persistent myelosuppression which was worsening day by day. He was supported with packed cell volume and platelet transfusions. On the fifth day, he was transferred to the intensive care unit for better monitoring. His platelet count increased to 45000/mm^3^ and WBCs to 1300/mm^3^ on the 10th day. His liver function deteriorated with bilirubin 8.1 grams on the 10th day. Unfortunately, he expired due to acute respiratory failure after six hours of onset on the 10th day.

### 2.2. Case 2

A 37-year-old male, a known case of psoriasis, presented with complaint of reduced oral intake due to painful lesions in the oral cavity, along with fever and chills for three days. He also complained of pain with ulceration in the existing lesions. On careful history taking, it was revealed that he took an unknown amount of oral MTX one week back without prior investigations.

**Figure 2:**
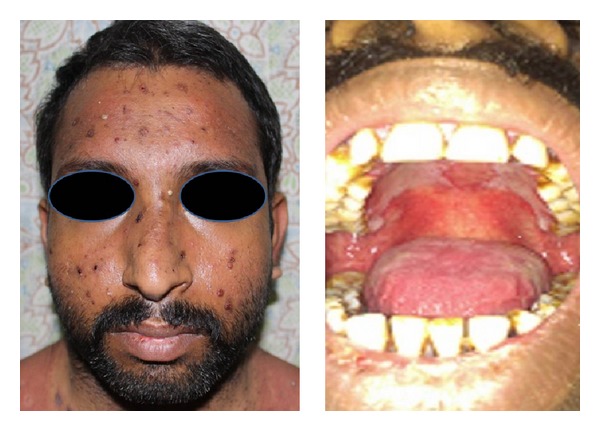
Pustules on face with mucositis in oral cavity (Case 2).

On examination, he was conscious but febrile with 102°F temperature with normal vitals. Cutaneous examination showed ulcerated and necrotic psoriatic plaques with erythema and tenderness. There were few new pustules on chest and face (Figure 2). He also had crusting and fissuring of the lips along with erosions in oral cavity. On admission, investigations suggested bone marrow suppression (hemoglobin 8.2 grams, WBC 1600 cells/mm^3^, and platelet count 1,06,000 cells/mm^3^) and altered renal functions (blood urea 72 and creatinine level 4.8).

Based on the clinical and laboratory findings, he was diagnosed having MTX toxicity and was covered with broad spectrum empirical antibiotics and injectable leucovorin. Sodium bicarbonate was added to aid in the excretion of drug by alkalinization of the urine and was hydrated aggressively. Despite aggressive therapy, there was gradual worsening with odynophagia/dysphagia and the blood counts still falling with no improvement in the renal functions. The patient was transferred to medicine ward. He was given platelet transfusions and taken for dialysis for two days, during which he succumbed to death.

## 3. Discussion

Low dose MTX in psoriasis rarely produces toxicity, and most of such cases occur due to failure to adhere to the recommended guidelines [1]. The risk of toxicity is greater if additional methotrexate is administered sooner than the usual scheduled weekly dose [3]. In the first case, it was a self-administration of the higher, consecutive dose which acted as a precipitating factor.

MTX toxicity has its impact on skin, gastrointestinal mucosa, liver, kidneys, and bone marrow. Ulcerations in skin due to MTX toxicity are restricted to the psoriatic plaques probably because of higher uptake of methotrexate by the hyperproliferative psoriatic plaques than normal skin [4]. Both of the cases presented with ulceration on existing plaques of psoriasis.

Pancytopenia due to MTX is attributed to the patients with renal dysfunction, presence of infection, folic acid deficiency, hypoalbuminemia, concomitant use of drugs such as trimethoprim, and advanced age [5]. Both of our patients had mucositis along with myelosuppression as a presenting feature of MTX toxicity. The probable cause of myelosuppression in the first patient could be advanced age, concomitant use of NSAID, and inadvertent use of MTX dose, while, in the second patient, it was renal dysfunction which was not picked up before initiation of the treatment.

Drugs can increase the risk of methotrexate toxicity either by decreasing renal elimination of methotrexate (aminoglycosides, cyclosporine, nonsteroidal anti-inflammatory agents, sulfonamides, probenecid, salicylates, penicillins, colchicines, cisplatin, and other renotoxic drugs) or by displacing methotrexate from protein binding sites in the plasma (salicylates, probenecid, sulfonamides, barbiturates, phenytoin, retinoids, sulfonylureas, and tetracyclines). NSAID taken for joint pain had contributed to the MTX toxicity in the first case.

Unfortunately, we could not measure the drug level of MTX because of lack of facility. But the common feature in both of them was inadvertent dosage of MTX which is the major contributory factor for the toxicity.

It is a must to avoid self-administration of such drugs. There should be proper counseling of the patient for not taking the drugs on their own without consulting a dermatologist as well as not to combine with any other drug without taking doctors' consent. Selling such drugs without prescription should be banned.

The second case had consumed MTX without following the standard investigative as well as therapeutic protocol. Already compromised renal functions were missed out and impairment in renal clearance could have played a role in MTX toxicity. Prior workup is mandatory for MTX which is otherwise really safe and effective in cases of psoriasis.

## Key Messages

Pretreatment investigations are a must if MTX is to be prescribed. Proper monitoring and strict avoidance of self-administration of MTX are mandatory. Coadministration of the drugs like NSAIDs should be judicious.
